# Clinical Outcomes of Early Versus Late Intubation in COVID-19 Patients

**DOI:** 10.7759/cureus.21669

**Published:** 2022-01-27

**Authors:** Ali Al-Tarbsheh, Woon Chong, Jozef Oweis, Biplab Saha, Paul Feustel, Annie Leamon, Amit Chopra

**Affiliations:** 1 Internal Medicine, Albany Medical Center, Albany, USA; 2 Pulmonary and Critical Care Medicine, Albany Medical Center, Albany, USA; 3 Pulmonary and Critical Care, Ozark Medical Center, West Plan, USA; 4 Neuroscience and Experimental Therapeutics, Albany Medical College, Albany, USA; 5 Internal Medicine, Albany Medical College, Albany, USA

**Keywords:** prolonged intubation, intubation complication, ventilation strategies, mechanical vent, covid-19

## Abstract

Background

The implications of intubation timing in COVID-19 patients remain highly debatable due to the scarcity of available evidence.

Objectives

Our study aims to assess the clinical characteristics and outcomes of COVID-19 patients undergoing early intubation compared to those undergoing late intubation.

Methods

This is a single-center retrospective study of adult COVID-19 patients admitted between March 1, 2020 and January 10, 2021. Early intubation was defined as intubation within 24 hours of a) hospital admission; b) respiratory status deterioration requiring FiO_2_ 60% and higher; or c) moderate/severe acute respiratory distress syndrome (ARDS) diagnosis.

Results

Among the 128 COVID-19 patients included, 66.4% required early intubation, and 33.6% required late intubation. The 28-day all-cause mortality and other outcomes of mechanical ventilation duration, hospital and ICU length of stay were equal regardless of intubation timing. Clinical characteristics, inflammatory markers, COVID-19 therapies, PaO_2_/FiO_2_ ratio, and pH were comparable for both groups. Better lung compliance was observed during early intubation than late intubation based on plateau (mean 21.3 vs. 25.5 cmH_2_O; P < 0.01) and peak pressure (mean 24.1 vs. 27.4 cmH_2_O; P = 0.04).

Conclusions

In critically ill COVID-19 patients, the timing of intubation was not significantly associated with poor clinical outcomes in the setting of matching clinical characteristics. More research is needed to determine which subset of patients may benefit from intubation and the predictors for optimal intubation timing.

## Introduction

Coronavirus disease 2019 (COVID-19) has a wide variety of clinical presentations from an asymptomatic carrier, upper respiratory tract illness, to severe respiratory failure in the form of acute respiratory distress syndrome (ARDS). During the COVID-19 pandemic, up to 35% of hospitalized patients will be transferred to the intensive care unit (ICU), and among the critically ill, around 15%-40% of patients will require invasive mechanical ventilation [1-3]. Initially, early intubation was advocated when COVID-19 patients demonstrated signs of impending respiratory failure without the option for non-invasive mechanical ventilation (NIV) in the form of continuous positive airway pressure (CPAP), bilevel positive airway pressure (BiPAP), and high-flow nasal cannula (HFNC). Concerns exist for the transmission of COVID-19 from patients to healthcare workers, particularly among those requiring aerosol-generating procedures and treatments, which includes NIV. However, in a similar fashion to NIV, endotracheal intubation itself can pose a similar risk for the aerosolization and transmission of COVID-19 to healthcare providers, even in a controlled setting [4]. Furthermore, early intubation of COVID-19 patients could result in unnecessary intubation and treatment in those who would have otherwise improved with a trial of NIV, in a medical resource-limited setting [5]. Other than the deficits in ventilators, the shortages of healthcare providers with appropriate expertise in managing the ventilators need to be considered.

As the wave of COVID-19 disease continues to spread across the globe resulting in multiple regional and large-scale outbreaks, respiratory support of NIV has been increasingly applied in an attempt to conserve depleting medical resources and delay or even avoid intubation in critically ill COVID-19 patients. The frequency of NIV use is around 5% in hospitalized COVID-19 patients but increases to 40% among critically ill patients [1,6-8]. Historically, although late intubation is associated with increased mortality among critically ill non-COVID-19 patients, it remains unclear if the same principle applies to COVID-19 patients with ARDS [9]. Wide variations in protocols exist at various medical institutions for the management of critically ill COVID-19 patients; however, there is no clear consensus on the timing of intubation or application of NIV trial before intubation. The purpose of our study is to assess the clinical characteristics and outcomes of critically ill COVID-19 patients who underwent early intubation compared to those receiving late intubation.

## Materials and methods

This study was a retrospective analysis of all critically ill adult patients diagnosed with COVID-19 admitted to the Albany Medical Center ICU, a large 745-bed regional tertiary care center located in Albany, New York, USA, between March 1, 2020 and January 10, 2021. The study was approved by the institutional review board of our institution and registered under protocol number 5825. Given the retrospective nature of our study, requirements for informed written consent were waived. COVID-19 was diagnosed by reverse transcription-polymerase chain reaction (RT-PCR) from a nasopharyngeal swab. Inclusion criteria were: a) COVID-19 patients age 18 years and above, and b) COVID-19 patients who required mechanical ventilation for acute respiratory failure during hospitalization. Acute respiratory failure was defined as a respiratory rate of more than 25 breaths per minute, bilateral pulmonary infiltrates on chest radiograph or computed tomography, and the need for 3 liters and higher oxygen therapy to maintain peripheral arterial oxygen saturation of 92% and above. The following COVID-19 patients were excluded: a) pregnant; b) incarcerated; c) intubation prior to hospital admission as a transfer from an outside hospital or in the field by emergency medical services in which the exact timing of intubation cannot be accurately verified; d) chronic tracheostomy or dependent on NIV or invasive mechanical ventilation due to underlying comorbidities (e.g., neuromuscular disease, obesity hypoventilation syndrome); and e) had an advance directive of “do not intubate” (DNI). The decision for ICU admission, oxygen therapy, respiratory support, and intubation was made at the discretion of the treating clinician. The clinician’s judgment was based on a multitude of factors, such as oxygen saturation, work of breathing, respiratory rate, mental status, and hemodynamics. We defined early intubation as COVID-19 patient who was intubated and mechanically ventilated within 24 hours of a) hospital admission; b) demonstrating a decline in respiratory status requiring 60% and more of fractional inspired oxygen (FiO_2_); or c) had moderate or severe degree of ARDS diagnosis, defined as the partial pressure of arterial oxygen and fractional inspired oxygen (PaO_2_/FiO_2_) ratio of less than 200. COVID-19 patients who did not meet the criteria for early intubation were categorized as delayed intubation. For COVID-19 patients who had multiple intubations during hospitalization, we included data from the first intubation period and excluded data from subsequent intubations. The total follow-up period was censored at 56 days from the time of admission for mechanically ventilated COVID-19 patients.

Data collection and outcomes

Data were extracted from the medical charts and electronic medical records. Patients with COVID-19 were identified using our institutional database, and patients requiring intubation were identified by ICD-10 billing Code (Z99.11) and comprehensive chart review. Patient confidentiality was protected by systematically deidentifying patients and storing patient data in a HIPAA-compliant institutional network drive that was password-protected and only accessible to those with an institution-associated account with permission given by the principal investigator. The clinical characteristics of COVID-19 patients were collected, which included: a) age; b) gender; c) ethnicity; d) body mass index (BMI); e) comorbidities; f) inflammatory markers upon admissions, such as ferritin, C-reactive protein (CRP), and D-dimer; and g) COVID-19 treatments such as corticosteroid, convalescent plasma, and remdesivir. We also collected the mean respiratory parameters on the day of initiation of invasive mechanical ventilation among COVID-19 patients were assessed involving: PaO_2_/FiO_2_ ratio; pH; tidal volume; tidal volume per kilogram; positive end-expiratory pressure (PEEP); plateau pressure; and peak pressure. Clinical outcomes of COVID-19 patients were gathered including a) 28-day all-cause hospital mortality; duration of invasive mechanical ventilation including tracheostomy; ICU and hospital length of stay (LOS). The primary purpose of our study was to determine the clinical indicators of 28-day all-cause in-hospital mortality; duration of invasive mechanical ventilation; ICU and hospital LOS. The secondary outcomes were to assess the clinical characteristics of COVID-19 patients receiving early versus delayed intubation.

Statistical analysis

Data were presented as mean with corresponding standard deviation for continuous variables, and numbers and percentages for categorical variables. Statistical inference for continuous variables was assessed by the Mann-Whitney test or student t-test, and Pearson’s chi-square test for categorical variables. Survival curves for COVID-19 patients undergoing early versus delayed intubation from inclusion to day 56 were developed using the Kaplan-Meier method with the log-rank test. All statistical tests were two-tailed and statistical significance was defined as P-value < 0.05. Analysis was performed using Minitab (v.19.2020.1) and R (v.3.6.1) statistical software.

## Results

**Figure 1:**
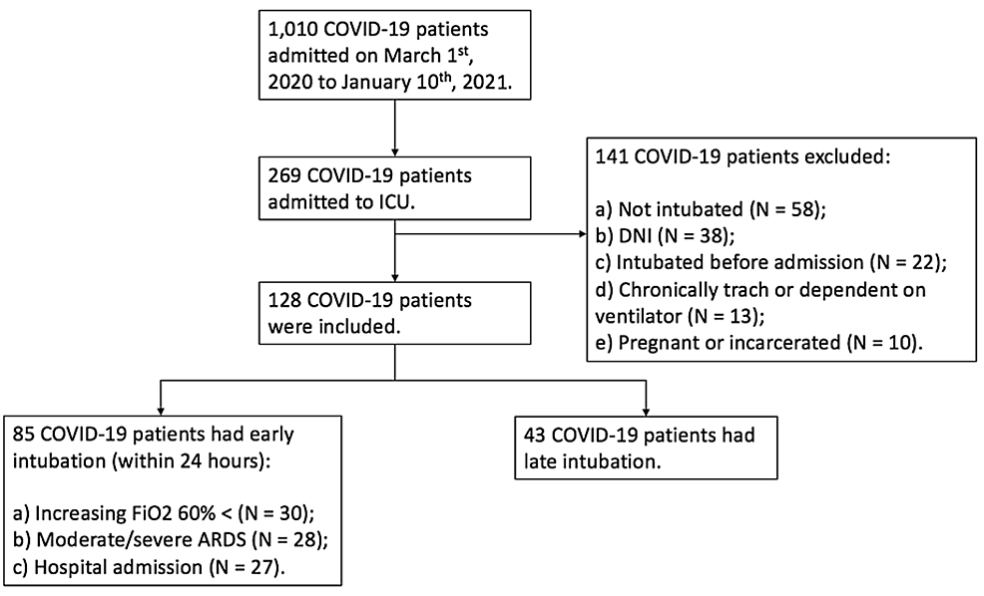
Flowchart for mechanically ventilated COVID-19 patients enrolled in the study.

A total of 1,010 patients who tested positive for COVID-19 was admitted to Albany Medical Center during the study period (Figure 1). Of these patients, 26.6% (269/1,010) required ICU care, out of 269 only 128 (47.5%) of patients met the inclusion criteria. 66.4% (85/128) had early intubation, and the remainder 33.6% (43/128) had late intubation. Among those who had early intubation, 35.3% (30/85) were intubated within 24 hours of increasing oxygen requirements above FiO_2_ 60%, 33.0% (28/85) within 24 hours of moderate or severe ARDS diagnosis, and 31.7% (27/85) were intubated within 24 hours of hospital admission. The mean age of COVID-19 patients requiring mechanical ventilation was 63.0 (+/- 14.1) years. We had an almost 2:1 male to female ratio (80/128), and the majority of patients (43.8% [56/128]) had a white ethnic background. Common chronic comorbidities observed were (56.3% [72/128]) hypertension, (41.4% [53/128]) diabetes mellitus, and (4.2% [31/128]) chronic pulmonary disease in which 75.0% (96/128) of patients had one and more comorbidities. Chronic pulmonary disease was defined as patients with underlying asthma, chronic obstructive pulmonary disease, bronchiectasis, and interstitial lung disease. The demographics and clinical characteristics were summarized in Table 1.

**Table 1:** Clinical characteristics of COVID-19 patients receiving early versus late intubation Abbreviations: BMI: body mass index; N: numbers; SD: standard deviations; Y: years.

Characteristics	Early intubation (N = 85)	Late intubation (N = 43)	P-value
Age (Y), mean [+/- SD]	62.0 [15.2]	66.0 [12.6]	0.11
Gender - N (%)			0.74
Male	54 (63.5%)	26 (60.5%)	
Female	31 (36.5%)	17 (39.5%)	
Ethnicity - N (%)			0.54
White	35 (41.2%)	21 (48.8%)	
Black	20 (23.5%)	13 (30.2%)	
Hispanic	7 (8.2%)	3 (6.9%)	
Asian	7 (8.2%)	1 (2.5%)	
Unknown	16 (18.8%)	5 (11.6%)	
BMI (kg/m^2^), mean [+/- SD]	29.3 [7.0]	32.1 [10.6]	0.12
Comorbidities - N (%)			
1 < Comorbidities	65 (76.5%)	31 (72.1%)	0.59
Chronic pulmonary disease	17 (20.0%)	14 (32.6%)	0.09
Diabetes mellitus	38 (44.7%)	15 (34.9%)	0.28
Coronary artery disease	17 (20.0%)	12 (27.9%)	0.32
Hypertension	47 (55.3%)	25 (58.1%)	0.76
Cancer	2 (2.4%)	1 (2.3%)	0.99
Admission Inflammatory markers, mean [+/- SD]			
Ferritin (ng/mL)	1,074.6 [586.0]	972.8 [621.0]	0.68
CRP (mg/L)	164.8 [156.0]	160.2 [156.5]	0.83
D-dimer (ng/mL)	14.2 [2.2]	8.6 [1.8]	0.17
COVID-19 treatments - N (%)			
Corticosteroid	75 (88.2%)	43 (100%)	0.09
Convalescent plasma	44 (51.8%)	29 (67.4%)	0.09
Remdesivir	20 (23.5%)	11 (25.6%)	0.80

The age difference between the early and late intubation group was comparable (mean 62.0 vs. 66.0 years; P = 0.11) (Table 1). There were no other significant demographic and comorbid differences between the two groups. On admission, the inflammatory markers of D-dimer (14.2 vs. 8.6 ng/mL; P = 0.17), ferritin (mean 1,074.6 vs. 972.8 ng/mL; P = 0.37), and CRP (mean 164.8 vs. 160.2 mg/L; P = 0.88) were similar. No significant differences were observed for COVID-19 treatments involving corticosteroid (88.2% vs. 100%; P = 0.09), convalescent plasma (51.8% vs. 67.4%; P = 0.09), and remdesivir (23.5% vs. 25.6%; P = 0.80) received by those requiring early and late intubation.

Regarding respiratory parameters on the day of intubation, the PaO_2_/FiO_2_ ratio (mean 146.3 vs. 173.0; P = 0.30) and pH (mean 7.3 vs. 7.3; P = 0.35) were comparable among those receiving early and late intubation (Table 2). No differences were demonstrated for ventilator parameters of tidal volume (mean 415.2 vs. 407.9 mL; P = 0.71), tidal volume per kg (mean 6.7 vs. 6.7 mL/kg; P = 0.86), and PEEP (mean 8.3 vs. 8.9 cmH_2_O; P = 0.37) upon intubation for both groups. COVID-19 patients in the early intubation group had better compliance with lower plateau (mean 21.3 vs. 25.5 cmH_2_O; P < 0.01) and peak pressure (mean 24.1 vs. 27.4 cmH_2_O; P = 0.04) than the late intubation group.

**Table 2:** Respiratory parameters of COVID-19 patients receiving early versus late intubation Abbreviations: N: numbers; PaO_2_/FiO_2_ ratio: partial pressure of arterial oxygen and fractional inspired oxygen; PEEP: positive end-expiratory pressure; SD: standard deviations.

Respiratory parameters on the day of intubation	Early intubation (N = 85)	Late intubation (N = 43)	P-value
PaO_2_/FiO_2_, mean [+/- SD]	146.3 [98.8]	173.0 [145.5]	0.30
pH, mean [+/- SD]	7.3 [0.12]	7.3 [0.14]	0.35
Tidal volume (mL), mean [+/- SD]	415.2 [63.8]	407.9 [71.9]	0.71
Tidal volume per kg (mL/kg), mean [+/- SD]	6.7 [1.4]	6.7 [1.3]	0.86
PEEP (cmH_2_O), mean [+/- SD]	8.3 [3.1]	8.9 [3.9]	0.37
Plateau pressure (cmH_2_O), mean [+/- SD]	21.3 [5.9]	25.5 [9.4]	< 0.01
Peak pressure (cmH_2_O), mean [+/- SD]	24.1 [7.2]	27.4 [9.4]	0.04

**Figure 2:**
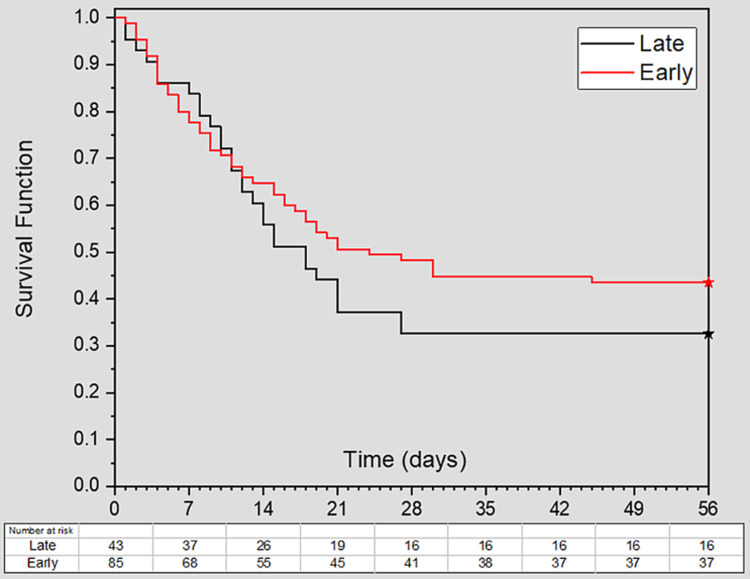
Kaplan-Meier survival curves for early and late intubations. Day zero is hospital admission, and living patients discharged from the hospital are censored at 56 days. There is no statistically significant difference between the curves (log-rank test, P = 0.28).

The clinical outcome of 28-day all-cause mortality was equal (56.5% vs. 67.4%; P = 0.23) for patients receiving early and late intubation with a pooled mortality rate of 60.2% (77/128). Figure 2 demonstrated the survival curves for both groups at day 56 from admission. There was no statistically significant difference between the curves (log-rank test, P = 0.28). The early intubation group had a similar duration of mechanical ventilation (mean 7.7 vs. 6.5 days; P = 0.28) than the late intubation group. ICU (mean 11.4 vs. 13.0 days; P = 0.19) and hospital LOS (mean 17.1 vs. 18.6 days; P = 0.44) were comparable for those in the early and late intubation group (Table 3).

**Table 3:** Clinical outcomes of COVID-19 patients receiving early versus late intubation Abbreviations: D: days; ICU: intensive care unit; LOS: length of stay; N: numbers; SD: standard deviations.

Outcomes	Early intubation (N = 85)	Late intubation (N = 43)	P-value
28-day all-cause mortality, N (%)	48 (56.5)	29 (67.4)	0.23
Duration of mechanical ventilation, (D) mean [+/- SD]	7.7 [7.35]	6.5 [6.0]	0.28
ICU LOS, (D) mean [+/- SD]	11.4 [8.8]	13.0 [9.4]	0.19
Hospital LOS, (D) mean [+/- SD]	17.1 [13.7]	18.6 [13.1]	0.44

## Discussion

Our study included 128 critically ill COVID-19 patients requiring mechanical ventilation, in which 66.4% required early intubation and the remainder 33.6% required late intubation. The overall mortality rate was 60.2%. Clinical characteristics of age, gender, BMI, ethnicity, comorbidities, and admission inflammatory markers were comparable to those requiring early and late intubation. No differences were demonstrated for COVID-19 therapies received during hospitalization or respiratory parameters of PaO_2_/FiO_2_ ratio and pH on the day of intubation in both groups. The early intubation group had a higher level of lung compliance, based on the higher plateau and peak pressure in the delayed intubation group, although other ventilatory parameters of tidal volume and PEEP were similar. The clinical outcomes of 28-day all-cause mortality, duration of mechanical ventilation, hospital and ICU LOS were equal regardless of the timing of intubation.

During the early stage of the pandemic, pre-emptive intubation was advocated by expert consensus to minimize the risk of viral transmission from patients to healthcare providers, prevent emergent intubation, and reduce patient self-induced lung injury (P-SILI) [10-12]. For reasons that in P-SILI, commonly seen in critically ill patients, involves vigorous spontaneous inspiratory efforts that can generate injurious transpulmonary pressure swings and is thought to parallel ventilator-induced lung injury (VILI) by augmenting the severity of ARDS [5,11]. In 2016, a large prospective cohort study demonstrated that delayed intubation was associated with an increase in mortality among critically ill non-COVID-19 patients (56% vs. 36%; P < 0.03) despite greater illness severity of patients in the early intubation group [9]. Furthermore, the early intubation group had a shorter duration of mechanical ICU LOS (9 days vs. 11.5 days; P < 0.01) and higher ventilator-free days (16 days vs. 7 days; P < 0.01). The idea that NIV only temporarily improves oxygenation and breathing in critically ill COVID-19 patients with respiratory failure without necessarily changing the natural course of the disease but poses a significant risk of viral transmission to healthcare providers is debatable. During the 2003 severe acute respiratory syndrome (SARS) epidemic, the use of NIV with adequate personal protective equipment was not shown to be associated with an increased risk of viral transmission to healthcare workers, whereas intubation, even in a controlled setting, increased the risk of viral transmission (RR 13.29; 95% CI 2.99-59.04; P < 0.01) [4]. In 2004, an observational study of SARS patients revealed that NIV prevented intubation in up to 70% of patients and was associated with shorter ICU LOS (3.1 days vs. 21.3 days; P < 0.01) than those requiring mechanical ventilation, without increasing viral transmission to healthcare workers [13]. Several observational studies revealed that the use of HFNC to manage acute respiratory failure in COVID-19 patients was associated with a lower rate of mechanical ventilation without significantly affecting mortality and ICU LOS in the setting of matching clinical characteristics and illness severity [14,15]. The early use of NIV involving CPAP and BiPAP for severe respiratory failure among 222 COVID-19 patients who shared similar clinical characteristics was associated with a reduction in mortality (OR 0.30; 95% CI 0.13-0.69; P < 0.01) compared to those requiring mechanical ventilation [16].

The difference in compliance, represented by plateau and peak pressure for COVID-19 patients receiving early versus late intubation, and the lack of improvement in mortality can be explained by the “atypical” phenotype of COVID-19-induced ARDS. This difference has been observed during the early course of the pandemic in which COVID-19 patients developed ARDS after seven days from initial infection, with a median of 11 (IQR 7-15) days from illness onset, and a high degree of lung compliance out of proportion to the degree of hypoxemia [2,10,17,18]. COVID-19 ARDS has a time-related disease spectrum with two primary phenotypes of type L and type H [17]. Early in the disease course (phenotype L), hypoxemia occurring in compliant lungs is due to the loss of lung perfusion regulation and hypoxic vasoconstriction, with a high shunt fraction [10,17]. Nevertheless, no improvement in oxygenation is observed despite high PEEP indicating a lack of poorly recruitable lungs, the usual mechanism in ARDS, but instead, respond to prone positioning due to the redistribution of perfusion in response to pressure and/or gravitational forces. Consequently, PEEP application early on in the disease course by intubation and mechanical ventilation is unlikely to improve gaseous exchange but may cause hyperinflation, worsen dead-space ventilation, and redirect blood flow away from overstretched well-ventilated airspaces while accentuating pre-existing microvascular injury [12]. This will further compromise O_2_ and CO_2_ gaseous exchange without the benefit of recruitment of functional lung volume. In the face of an ongoing right-to-left shunt, the lack of respiratory distress and a compensatory increase in ventilation among COVID-19 patients is due to the low PaCO_2_ level [19]. The respiratory centers are more sensitive to changes in partial pressure of arterial carbon dioxide (PaCO_2_), where minimal changes in PaCO_2_ will outweigh PaO_2_ and blunt the respiratory drive [20]. Over time, type L COVID-19 patients may improve, remain stable, or worsen and develop type H. Type H phenotype is defined as high elastance (low compliance) due to progression of COVID-19 ARDS severity with increased lung permeability and edema from inflammation [17]. Finally, respiratory distress will occur from activation of both hypoxic and hypercapnic ventilatory drive in the respiratory center due to lung edema reaching a certain magnitude resulting in dead-space ventilation with PaCO_2_ retention [17,19,20]. Hence, mechanical ventilation is eventually warranted as a last resort due to the decline in respiratory status. In our study, no clinical significance was observed when intubating COVID-19 patients with one phenotype versus another.

Several retrospective observational studies have been published assessing the clinical implications of early versus late intubation among critically ill COVID-19 patients. The earliest observational study evaluating the timing of intubation was a multi-center study by Lee et al. conducted between February and April 2020, where early intubation was defined as intubation within 24 hours of meeting ARDS criteria [21]. No difference in clinical characteristics and outcomes of mortality, duration of mechanical ventilation, ICU LOS, and incidence of ventilator-associated pneumonia (VAP) was observed for the 39 COVID-19 patients receiving early versus late intubation. Several single-center observational studies conducted between March and May 2020 by Matta et al. and Siempos et al. revealed that clinical outcomes were comparable in COVID-19 patients, regardless of their respective timing of intubation, although, in the study by Matta et al., those requiring early intubation had greater disease severity based on Sequential Organ Failure Assessment (SOFA) score [5,22]. In those studies, early intubation was defined as intubation upon admission or within 1-2 days of requiring a higher level of oxygen support (FiO2 50% and higher) by means of NIV. A single-center retrospective study by Pandya et al. was the only study that demonstrated a shorter ICU and hospital LOS in the setting of matching clinical characteristics among COVID-19 patients receiving early intubation during the first wave of the pandemic; however, the mortality rate did not differ compared to the late intubation group [23]. The mean time to intubation from admission in the early intubation group was 0.2 (+/- 0.3) days compared to 5.5 (+/- 5.0) days in the delayed intubation group. In our study, the early intubation cohort had a lower PaO2/FiO2 ratio, although not statistically significant, suggesting an increased severity of lung injury. Nevertheless, the comparable clinical outcomes in this setting support the notion that delaying intubation is not harmful to COVID-19 patients.

The strengths of our study include the detailed comparison of clinical characteristics, including inflammatory markers and respiratory parameters of mechanically ventilated COVID-19 patients that are important markers for illness severity. Clinical outcomes such as 28-day mortality, duration of mechanical ventilation, ICU and hospital LOS that have important implications on patients’ prognosis and healthcare resources were assessed. Moreover, patients with advance directives of DNI were excluded from the study so that differences in goals of care and treatment provided would not trigger any bias to the study results. To assess the clinical outcomes in a more pragmatic manner that represents challenges commonly face in the current critical care environment, we included a greater proportion of COVID-19 patients that we strongly believed represent the early intubation group as defined in our inclusion criteria. This decision is to improve generalizability based on evidence that it is not uncommon for COVID-19 patients who require ICU admission and ultimately mechanical ventilation to experience a decline in respiratory status during a median of 12 (IQR 7-16) days of illness onset [2,18]. The generalizability of our study is further enhanced by the broad duration of enrollment between March 2020 and January 2021 compared to other retrospective studies that evaluated COVID-19 patients during the first wave of COVID-19 outbreak from February to May 2020 [5,21-23]. The prolonged duration of follow-up of 60 days from admission will prevent length time bias from the overestimation of survival among mechanically ventilated COVID-19 patients as the median hospital and ICU LOS is 17 (IQR 10-22) days and 8 (IQR 5-13) days, respectively [18,24]. Moreover, both the early and late intubation groups received similar COVID-19 therapies such as corticosteroids and remdesivir, which have since been the standard of care with proven efficacy in reducing mortality and the need for mechanical ventilation among hospitalized COVID-19 patients [25,26].

There were several limitations to our study that warrant discussion. First, this was an observational retrospective study involving a single tertiary care center that included 128 critically ill patients. Therefore, the lack of difference in outcomes of all-cause mortality, duration of mechanical ventilation, and ICU and hospital LOS could be due to the modest sample size, which would limit the statistical power for the detection of small differences in the outcomes measured. Furthermore, the results of our study could not be extrapolated to other ICU from different regions or countries with varying protocols and treatment algorithms for the management of critically ill COVID-19 patients. Nevertheless, this was the case for several other studies [5,22,23]. Second, the prolonged enrollment period between March 2020 and January 2021, combined with the retrospective nature of data collection and changes in guidelines at different periods of enrollment due to the rapid advancement of evidence-based medicine for COVID-19 would likely predispose to a confounding bias. Third, although indications of intubation were explained, detailed data on the exact timing of intubation were not available as those data were inconsistently recorded and often relied on the judgment of the treating clinician. The data for the timing of intubation were collected from orders placed in the EMR. However, it is likely, given the strains placed on healthcare providers in the context of the pandemic conditions, the ordered times do not accurately reflect the time of intubation. Specific data on heart and respiratory rate was not collected, which could indicate the reasons for intubation. Although respiratory variables involving arterial blood gas (ABG) and ventilator parameters were obtained during the day of intubation, further data on the serial progression of ABGs and ventilator parameters during the period of mechanical ventilation were not assessed, which could be valuable prognostic indicators for illness severity and death. Fourth, although certain serum inflammatory markers were examined during hospitalization, other inflammatory markers could be important prognostic indicators such as white cell counts, lymphocyte counts, erythrocyte sedimentation rate (ESR), lactate dehydrogenase (LDH), and fibrinogen were not assessed [27,28]. Fifth, important clinical scoring systems to determine the severity of illness, such as SOFA and Acute Physiological Chronic Health Evaluation (APACHE II) score, were not assessed in our study, which might indicate the reason for early intubation and mechanical ventilation. Sixth, the type and duration of respiratory support received, such as conventional oxygen therapy or NIV involving HFNC, CPAP, and BiPAP, were not assessed before intubation events as NIV had been demonstrated to be a valuable tool for decreasing the need for mechanical ventilation without significantly affecting mortality [14,15,29]. Lastly, other important outcomes that have important implications in patients’ care and healthcare resources, such as the incidence of VAP, the need for tracheostomy, renal replacement therapy (RRT), and extracorporeal membrane oxygenation (ECMO) support, were not assessed in our study.

A large, well-designed multi-center prospective study is required to determine the clinical implications of the timing of intubation among COVID-19 patients with detailed data on the clinical characteristics, which includes serum inflammatory markers, illness severity based on SOFA and APACHE II scores, and respiratory parameters before and during the period of mechanical ventilation. Future studies should be focused on: 1) how the different clinical characteristics and illness severity of critically ill COVID-19 patients may affect the timing of intubation and the subsequent outcomes of mortality, ICU and hospital LOS; 2) whether the use of NIV respiratory support is a safe and effective method to reduce the need for mechanical ventilation; 3) in a similar fashion as corticosteroids, the role of other immunomodulators or antiviral agents as life-saving therapy in decreasing the requirement of mechanical ventilation; 4) can lung-protective and recruitment strategies improve survival rate and shorten the duration of mechanical ventilation; 5) the incidence of VAP and the need for tracheostomy, RRT, and ECMO support among COVID-19 patients based on the timing of intubation; and 6) long-term outcomes, such as pulmonary function test and quality of life after discharge 7) isolation and identification of local infectious agents such as biofilm-forming bacteria and fungi.

## Conclusions

In critically ill COVID-19 patients, the timing of intubation was not significantly associated with poor clinical outcomes and was mainly driven by the progression of clinical status. Early in the disease course, the atypical phenotype of COVID-19-induced ARDS with severe hypoxemia from loss of lung perfusion regulation and hypoxic vasoconstriction, in the setting compliant lungs, may explain the lack of benefit from early intubation. Hence, early intubation is not always beneficial for COVID-19 patients and may cause a delay in providing life-saving treatment for other critically ill patients in a medical resource-limited setting. It may even be clinically prudent to use intubation as a last resort and consider NIV when COVID-19 patients develop a deterioration in respiratory status. The factors highlighted in our study will provide some guidance for future prospective study planning. More research is needed to determine which subset of patients may benefit from intubation and the predictors for optimal timing of performing intubation. The results of our study have significant implications on patient selection and decision-making in the allocation of limited medical resources, such as mechanical ventilators.
